# Fishing for Florida Bass in West Virginia: Genomic Evaluation of Florida Bass Presence and Establishing Baselines of Genetic Structure and Diversity for Native Largemouth Bass

**DOI:** 10.3390/biology14040392

**Published:** 2025-04-09

**Authors:** Andrew Johnson, Katherine Zipfel, Dustin Smith, Amy Welsh

**Affiliations:** 1 School of Natural Resources and the Environment, West Virginia University, Morgantown, WV 26506, USA; amy.welsh@mail.wvu.edu; 2 West Virginia Division of Natural Resources, 324 4th Avenue, South Charleston, WV 25303, USA; katherine.j.zipfel@wv.gov (K.Z.); dustin.m.smith@wv.gov (D.S.)

**Keywords:** Florida bass, largemouth bass, fisheries, non-native species management

## Abstract

Florida bass (*Micropterus salmoides*) and largemouth bass (*Micropterus nigricans*) are iconic sport fish that hybridize readily, influencing fishery management practices. While the Florida bass has been introduced to various U.S. states to create trophy fisheries, its genetic introgression into native populations can lead to ecological and genetic consequences. Recognizing the need to assess Florida bass presence to guide future management directions, diagnostic SNPs were genotyped for 856 putative largemouth bass across 31 sampling locations across the state of West Virginia. Florida bass controls and a reduced representative sample of 226 individuals from 19 sampling locations were sequenced using the genotype-by-sequencing dd-RAD protocol. The results from the two genomic investigations found no Florida bass ancestry in West Virginia populations, suggesting either no introduction or failed reproductive success of Florida bass in the state. Among West Virginia largemouth bass populations, unique genetic ancestries were found predominantly in introduced non-native largemouth bass populations, indicating that the only sub-structuring in the state is a result of stocking non-native ancestries into the state. Genomic diversity was found to be higher in Ohio River pools compared to inland reservoirs, as well as showing higher levels of potential inbreeding. These results underscore the need to preserve the genetic integrity of native Ohio River strain largemouth bass and prevent the introduction of the Florida bass or F_1_ hybrids into the Ohio River and other watersheds of West Virginia. Management recommendations include prioritizing the stocking of native strain bass to mitigate inbreeding and avoid introducing Florida bass to conserve genetic diversity.

## Simple Summary

Florida bass are often stocked into largemouth bass populations in an attempt to create a trophy fishery due to the potential for enhanced growth. Largemouth bass populations were sampled from across the state of West Virginia and genotyped using two different genomic techniques to quantify the presence of Florida bass alleles and ancestry in the state. Using the two techniques, no presence of Florida bass ancestry was detected, highlighting either a lack of introduction or failed reproduction. Future management directions should emphasize stocking the native strain and avoid introducing Florida bass to conserve native genetic diversity.

## 1. Introduction

Florida bass (*Micropterus salmoides*) and largemouth bass (*Micropterus nigricans*) are iconic freshwater sport fishes in North America and represent two distinct species complexes that are known to readily hybridize [1,2,3]. Despite similar phenotypic appearances the species display different growth dynamics, with the Florida bass and the F_1_ hybrid of the two species known to have the potential to reach larger sizes at later life stages in warmer climates compared to the northern largemouth bass [4,5]. With management for largemouth bass population dynamics largely focused on management for larger individuals, Florida bass and the F_1_ hybrid have been routinely stocked outside of their native range as a management tool in attempts to enhance recreational fisheries and create trophy fishery opportunities [5,6,7].

The early stocking efforts of Florida bass across the United States are well documented; shortly after the description of the Florida bass in 1949, California began stocking Florida bass into their state [8,9]. Due to the difficulty in morphologically distinguishing the two species [10], genetic testing is often employed to differentiate the two species and assess the level of introgression of the two species within populations [2,11,12,13]. The increasing affordability of next-generation sequencing has allowed for the development of diagnostic single-nucleotide polymorphism (SNP) panels, with fixed SNP differences between the two species being readily available to investigate the prevalence of Florida bass ancestry within largemouth bass populations at a relatively low cost [2,12,14,15,16].

This utilization of Florida bass as a fisheries management tool and the introduction of Florida bass outside of its native range has led to the introgression of Florida bass alleles into native largemouth bass gene pools, with this introgression having been observed in Texas, Tennessee, Arkansas, Alabama, Oklahoma, and Louisiana [2,5,6,7,11,17,18]. The introgression of non-native alleles into a gene pool poses potential detrimental consequences on that native population including outbreeding depression, loss of locally adapted traits, change in allele frequencies in local populations, and overall reduction in the fitness of the native population [19,20,21,22]. While the introgression of non-native alleles poses risks on a molecular level, the risk of Florida bass outcompeting largemouth bass due to their larger size may compound these issues on native communities and ecosystems to a greater extent [13]. Their potential larger size can be viewed as beneficial from a recreational fisheries perspective, with some state management agencies stocking Florida bass and F_1_ hybrids to attempt to increase growth rates of the native population in an effort to create an enhanced trophy fishery for anglers [5,16,23]. However, the introduction of non-native species and resulting introgression of non-native alleles can lead to negative impacts on locally adapted gene complexes and a loss of local genetic diversity [24,25].

While many studies have been implemented to investigate the effects on a population following the stocking of Florida bass into a system, comparatively few studies have been performed to assess the levels of Florida bass ancestry in different populations prior to stocking [15]. Florida bass alleles are often unexpectedly found in systems in which they are non-native to as a result of stocking, including in Oklahoma, Tennessee, Louisiana, and Texas [5,15]. Recognizing the importance of assessing population-level ancestry and the desire from some anglers to have F_1_ hybrid bass stocked in the region to enhance trophy fisheries opportunities, the West Virgina Division of Natural Resources (WVDNR) sampled established largemouth bass fisheries throughout the state to determine if Florida bass or their hybrids had been introduced into the state and their potential prevalence within the state. Identifying fisheries that may have already had Florida bass introduced was essential to help guide future decisions on the management of black bass fisheries in West Virginia.

The present study consists of three distinct objectives utilizing two genomic approaches that will assist the WVDNR in creating best management practices for largemouth bass fisheries throughout the state. First, Genotyping-in-Thousands by sequencing (GT-seq) was utilized using known fixed SNPs between the Florida bass and northern largemouth bass to quantify the extent of Florida bass allele prevalence in all sampled largemouth bass throughout the state. This approach will identify if any systems may already have a higher prevalence of Florida bass alleles, as well as identify systems that need to be conserved due to a low prevalence or the absence of Florida bass ancestry. Secondly, known Florida bass and putative F_1_ hybrids between the two species were also analyzed using a genotype-by-sequencing (GBS) approach utilizing double-digest restriction-site associated DNA sequencing (ddRAD-seq) to supplement the GT-seq approach, with datasets generated to directly compare known Florida bass to West Virginia largemouth bass populations using a greater number of SNPs. Lastly, largemouth bass from various populations throughout the state were sequenced to investigate the population structuring of largemouth bass within West Virginia, quantify the genomic diversity of populations throughout the state to create a genomic diversity baseline, and identify potential populations of management concern.

## 2. Materials and Methods

### 2.1. Sample Collection

**Figure 1:**
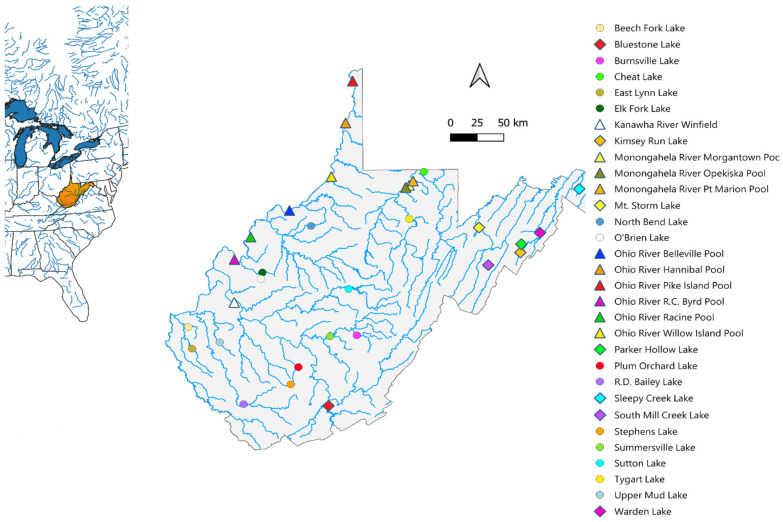
A map of eastern North America and major river systems is shown on the left, with the extent of West Virginia highlighted in orange. Sample locations across West Virginia are shown on the right, with river sampling locations indicated by triangle sample points. Eastern sampling locations are indicated by a diamond-shaped point, where largemouth bass are considered an introduced species.

Comprehensive sampling of largemouth bass throughout the state of West Virginia was conducted, resulting in 899 individual samples being collected from 31 different sampling locations between 2020 and 2024 (Figure 1). All sampling was performed by the West Virginia Division of Natural Resources (WVDNR) following a standard protocol. Fin clips were taken from individual largemouth bass and sent to the Wild Genomics Lab at West Virginia University for genetic analysis. Four known Florida bass (*n* = 3, Lake Walk-in-Water, FL; *n* = 1, Lake Yale, FL) and three putative F_1_ hybrids (*n* = 2, Millers Creek Reservoir, TX; *n* = 1, John Paul Landing Reservoir, TX) were also obtained from the Texas Parks and Wildlife to verify the diagnostic SNPs and were sequenced to directly compare to West Virginia largemouth bass populations.

### 2.2. GT-Seq Laboratory Methods

DNA was extracted from fin clips using the QIAcube HT^®^ automated nucleic acid purification instrument using the QIAamp 96 DNA QIAcube HT Kit (©Qiagen, Hilden, Germany). DNA extracts were quantified on a Nanodrop spectrophotometer (©ThermoScientific, Wilmington, DE, USA) and standardized to a concentration of 20 ng/µL for downstream protocols.

To accurately assign individual bass ancestry, 25 known diagnostic SNPs between largemouth bass and Florida bass were assessed in a GT-seq approach [12,26]. A master mix consisting of standardized DNA, Qiagen Plus multiplex master mix (2X), and the 25 species-specific primers in a 7 μL mixture per well were used for PCR amplification with thermocycling conditions set at 95 °C for 15 min, 35 cycles (95 °C 30 s, 60 °C 90 s, 72 °C 30 s), 68 °C 10 min., and a 4 °C hold. PCR product was then diluted, with 3 μL of PCR product being added to 37 μL of nuclease-free water. Diluted PCR product from 8 random samples from each plate was amplified on a 1% agarose gel to confirm the presence of DNA and validate the first PCR step. Unique i05 barcodes were added to each well to distinguish individuals, and a distinguishing i07 barcode was added to each plate to differentiate plates upon pooling. The i05 and i07 barcodes, PCR product, and Qiagen Plus multiplex master mix were then added to create a 10 μL mixture that underwent PCR amplification with thermocycling conditions as follows: 95 °C 15 min, 10 cycles (95 °C 10 s, 65 °C 30 s, 72 °C 30 s), 72 °C for 5 min. with a 4 °C hold. Following this step, each plate of samples was normalized using the SequalPrep™ Normalization Plate Kit (Applied Biosystems, Thermo Fisher Scientific, Waltham, MA, USA) following the manufacturer’s protocol. Samples in each plate were then pooled and purified using the GeneJET PCR Purification kit (©Thermo Fisher Scientific, Waltham, MA, USA) following the manufacturer’s protocol, with the final elution step set to 30 μL. Purified PCR product from each plate was then run on a Pippin Prep (©Sage Science Beverly, Beverly, MA, USA) following the manufacturer’s protocol, with the target size range being set to 200–300 bp. The resulting product was then sent to the West Virginia University Genomics Core facility for sequencing on an Illumina MiSeq (©Illumina, San Diego, CA, USA).

### 2.3. ddRAD-Seq Genotype-by-Sequencing Laboratory Methods

Standardized genomic DNA was processed using a double -digest restriction-site-associated DNA sequencing (ddRAD-seq) genotype-by-sequencing (GBS) protocol [27]. In total, 10 μL of standardized genomic DNA was added to a master mix containing 16.2 μL of nuclease-free water, 3.0 μL of 10X NEBuffer4 (New England Biolabs, Ipswich, MA, USA), and 0.4 μL of PstI and MspI restriction enzymes (New England Biolabs #R3140L and #R0106L, respectively) and run with thermocycler conditions set to 37 °C for 120 min. and 80 °C for 20 min. with an infinite hold of 8 °C. A master mix containing 8.4 μL of nanopure water, 5.0 μL of 10X T4 DNA Ligase Reaction Buffer, and 1.6 μL of T4 DNA Ligase (New England Biolabs #B0202S and #M0202L, respectively) per sample and 5 μL of unique individual barcodes was added to each well and ligated to the resulting DNA fragments (thermocycler conditions set to 22 °C for 120 min. and 65 °C for 20 min. with an infinite 8 °C hold). Samples were then pooled and purified using the GeneJET PCR Purification Kit (©ThermoScientific, Wilmington, DE, USA) following the manufacturer’s standard protocol, with the purified samples run on a Pippin Prep (Sage Science, Beverly, MA, USA) to collect fragments between 250 and 450 basepairs in length. In total, 2 μL of the resulting product was added to a master mix containing 22.5 μL of nanopure water, 10 μL of 5X Q5 buffer, 10 μL 5X Q5 High GC Enhancer, 0.50 μL Q5 High-Fidelity DNA Polymerase (2000 units/mL) (New England Biolabs MO491L), 2 μL of both forward and reverse Illumina primers at 10 μM, and amplified using the following parameters: 5 min. at 72 °C, 30 s. at 98 °C, 21 cycles (10 s. at 98 °C, 30 s. at 65 °C, and 30 s. at 72 °C), a final extension of 5 min. at 72 °C, and an infinite hold of 4 °C. The resulting PCR product was then purified again using the GeneJET PCR Purification Kit (©ThermoScientific, Wilmington, DE, USA) and eluted to a final volume of 50 μL. The quality and quantity of the generated libraries were assessed using an Agilent 2100 BioAnalyzer (Agilent Technologies, Santa Clara, CA, USA) at the West Virginia University Genomics Core Facility. The generated and quality control validated libraries were then sequenced on an Illumina NextSeq2000 (Illumina, San Diego, CA, USA) at the Marshall University Genomics Core Facility.

### 2.4. GT-Seq Analysis

To identify the genomic position and verify the fixed SNPs in the 25 SNP panel [12], generated raw sequences were compared between known Florida bass (*n* = 4) and previously identified largemouth bass from Sutton Lake (*n* = 21) that were identified using diagnostic SNPs between black basses but excluded Florida bass [28]. Verified SNPs were then scored using the GTscore pipeline (https://github.com/gjmckinney/GTscore, accessed on 9 October 2024) [22] on all sampled largemouth bass. Loci with low coverage (<150X) and individuals with one or more missing loci were removed from the study. Individuals were scored for the presence of native northern largemouth bass ancestry at each SNP, with a homozygous call for the largemouth bass allele given 100% ancestry, a homozygous call for the Florida bass allele given 0%, and a heterozygous call given 50% largemouth bass ancestry. Individual largemouth bass ancestry was then calculated for each individual, and population-level averages of largemouth bass ancestry were calculated for each sampling site.

NEWHYBRIDS ver. 1.1 [29] was used with the EasyParallel gui [30] to classify individuals into six genotype hybrid classifications: pure Largemouth bass, pure Florida bass, F1 hybrid, F2 hybrid, an F1 backcrossed with a pure Florida bass (FB_Bx), and an F1 backcrossed with a pure Largemouth bass (LMB_Bx) The dataset was analyzed using 10,000 burn-in and 10,000 sweeps over six runs. Individuals that displayed > 80% probability of belonging to a genotype classification were assigned to that classification, while individuals with <80% probability were scored using the two highest genotype classifications with the first classification indicating higher probability. For example, an individual with 75% probability of being a pure largemouth bass and 25% probability of being an F2 hybrid would be classified as an LMB/F2 hybrid, as would an individual with 51% and 49%, respectively. The prevalence of individual genotype classifications for each of the sampled populations was assessed to further ascertain population-level genetic ancestry.

### 2.5. ddRAD-Seq Genotype-by-Sequencing Analysis

Generated multiplexed sequence libraries were first processed using the CUTADAPT program ver. 5.0 [31] to remove adaptor sequences and discard reads that were shorter than 50 bp in length. The filtered libraries were then processed using the STACKS software program ver. 2.65 [32]. *Process_radtags* was used to demultiplex the data and separate individuals by barcode sequences. Sequences were cleaned (-c), and low-quality reads were discarded (-q), with low-quality reads being discarded if bases were uncalled or if the raw phred score dropped below 10 using a sliding window of 15%.

Paired-end sequences for each individual were aligned to the largemouth bass reference genome (NCBI WGS accession: JAKUMD01) [33] using Bowtie 2 software ver 2.5.0 [34], and the resulting SAM files were converted to BAM files using SAMtools software ver 1.21 [35]. Aligned sequences were then processed using the STACKS *ref_map* pipeline. The number of reads generated, mean coverage, and number of loci generated for each individual were assessed using the gstacks.log.distribs file generated following the *ref_map* pipeline. Individuals with low coverage (<3x) and a low number of loci (<50,000 loci) were removed from downstream analyses. The resulting catalogs were then processed and filtered using the *populations* program with the following parameters: To be filtered on the final dataset, a locus had to be found in every population and present in 80% of the individuals within each of the populations; only the first SNP for each locus was used for population genetics investigations (--write-single-snp) to mitigate the influence of linked SNPs in genomic population structuring assessments. A minor allele count filter (--min-mac) of 3 was used to eliminate singletons and doubletons and remove errant genotyping calls from the dataset. The final genomic dataset was exported into genepop [36] and PLINK [37] formats for genomic analysis.

### 2.6. Population Structuring and Genomic Diversity Analysis

The exported SNP dataset was converted to binary file formats using plink [37], and SNP data management and analyses were performed using wrapper functions within the R package SambaR ver. 1.10 [38] (GitHub page: https://github.com/mennodejong1986/SambaR, accessed on 16 October 2024). Population structuring was assessed using various models. Initial population structuring inferences were carried out using principal component analysis (PCA) using PLINK ver. 1.9 (--pca) [37] and by plotting the resulting eigenvectors for each individual using ggplot2 ver. 3.5.1 [39]. Population structuring inferences were assessed using discriminant analysis of principal components (DAPC) in the R package adegenet ver. 2.1.10 [40]. k-means clustering was performed after the data were transformed into a PCA in adegenet [40], with 1–40 clusters tested. DAPC was then performed in adegenet [40] and analyzed on the transformed data, with the number of clusters being defined by the k-means Bayesian Information Criterion (BIC) score. Sparse nonnegative matrix factorization (snmf) in the R package LEA ver. 3.16.0 [41] was also used to assess the number of ancestral populations, where the optimal number of clusters was determined using the elbow method on cross-entropy scores generated using the LEAce function within SambaR, which generates cross-entropy scores over a defined number of runs for a defined number of K values (50 runs and K 1–12 were tested). Pairwise population differentiation (F_ST_) was assessed between sampled populations using the Weir and Cockerham equation [42], with heatmaps generated using the calcdivergence function in SambaR [38].

Genomic diversity estimates for observed and expected heterozygosity (H_O_ and H_E_), nucleotide diversity (π), the number and proportion of SNPs out of Hardy–Weinberg equilibrium (HWE), the proportion of polymorphic loci, the mean frequency of the most frequent allele at each locus (P), and inbreeding coefficients (F_IS_) for each population were generated from the STACKS population output [32]. The number of private alleles within each population and Watterson’s Θ [43] and Tajima’s D [44] per nucleotide were assessed using the calcdiversity function within SambaR [38]. Selection analysis was performed to identify putatively adapted SNPs using OutFLANK-0.2 [45] and pcadapt-4.3.5 [46], with only SNPs that were identified in both approaches categorized as potentially undergoing selection. Effective population sizes (N_e_) for all sampled populations were calculated using the R package dartR ver. 2.9.7 using the gl.LDNe function, which utilizes the NeEstimator software ver. 2.1 [47,48]. All effective population size estimates used a threshold allele frequency of 0.05.

## 3. Results

### 3.1. Dataset Generation

Of the 25 original SNPs used in the panel, 16 were retained after being validated between known Florida bass and largemouth bass (Table 1) and displaying sufficient coverage (>150X) (Table 2). After the removal of individuals that had one or more missing SNPs, 856 largemouth bass from all 31 sampling locations within West Virginia were retained along with 4 known Florida bass and 3 putative F1 hybrids. During validation, all 4 Florida bass were homozygous for the Florida bass allele at each of the 16 diagnostic SNPs except for 1 individual from Lake Walk-in-Water, which was heterozygous at SNP 19,092. Of the 20 previously identified largemouth bass used during validation, 5 were heterozygous at SNP 8751, 1 individual was homozygous for the Florida bass allele, and 1 largemouth bass was heterozygous at SNP 31,992. All other SNPs among the 20 largemouth bass and 4 Florida bass were homozygous for their respective diagnostic SNPs.

Following the removal of individuals with poor sequencing coverage and a low number of loci, two different ddRAD-seq datasets were generated. The first dataset consisted of 226 largemouth bass from 19 different sampling locations within West Virginia and a population that consisted of the 4 known Florida bass and 3 putative F1 hybrids. The resulting dataset consisted of 2772 SNPs and a genotyping rate of 96.11%. A second dataset excluding the known Florida bass and putative hybrids consisted of 2618 SNPs with a genotyping rate of 95.67%. After removing individuals and populations found to be of the non-dominant genetic ancestry, a final dataset consisting of 3599 SNPs with a genotyping rate of 95.41% was generated that contained 153 individuals from 13 sampling locations to quantify the genomic diversity of the native strain of West Virginia largemouth bass. Largemouth bass from the Hannibal and Pike Island pools of the Ohio River were combined together due to the low number of individuals in each pool and because they are neighboring pools within the Ohio River system.

### 3.2. GT-Seq Ancestry Results

All four known Florida bass genotyped in the present study displayed a 99.999% probability of being pure Florida bass. Among the three putative F1 hybrids, one displayed a 99.97% probability of being a pure Florida bass, the second displayed a 91.39% probability of being a pure Florida bass, and the last individual displayed a 59.1% probability of being an F1 hybrid and a 33.52% probability of being an F1 backcrossed with a Florida bass.

Of the 856 largemouth bass sampled across West Virginia, 760 had >80% probability of being classified as pure largemouth bass, with 727 of these displaying a >95% probability of being a pure largemouth bass (Table 3). The second most prevalent genotype classification was an F_1_ backcrossed with a largemouth bass (LMB_Bx), with 62 individuals classified as this genotype. This genotype classification was most commonly found in East Lynn Lake (*n* = 13), the Monongahela River (*n* = 11), Sleepy Creek Lake (*n* = 8), Upper Mud Lake (*n* = 6), and Bluestone Lake (*n* = 5). One individual was classified as an F_2_ hybrid from Sleepy Creek Lake with a probability of 82.4%. The remaining 33 individuals did not meet the 80% threshold for any of the six classifications. Of these 33, 19 had a range of 52.9–79.3% probability of classifying as the pure largemouth bass genotype with LMB_Bx being the second highest genotype classification probability for all 19 individuals. In total, 12 individuals had a range of 50.1–78.5% probability of being LMB_Bx, with the largemouth bass genotype being the second highest classification for all 12 of these individuals. One individual from Sleepy Creek Lake had a 48.4% probability of being LMB_Bx with the second highest probability being an F2 hybrid at 24.5%, and the last individual from East Lynn Lake had a 37.9% probability of being an F1 hybrid as its highest genotype classification probability, with F2 hybrid being the second highest genotype classification at 29.0%.

Paralleling the NEWHYBRIDS results, East Lynn Lake and Sleepy Creek Lake both display the lowest average largemouth bass ancestry among all populations with an average of 86.5% and 84.8%, respectively, and they were the only two sampling locations to have an average and median ancestry below 90%. When looking at the state of West Virginia as a whole, the average largemouth bass ancestry was 94.92%, with an average of 1.86 loci displaying heterozygosity per individual on a statewide basis. In total, 278 (32.5%) of the tested West Virgina largemouth bass contained 100% largemouth bass ancestry at each of the 16 tested SNPs. Sutton Lake (65.2%), Monongahela River Pt. Marion Pool (61.7%), Warden Lake (60.9%), Opekiska Pool (55.2%), and Morgantown Pool (55.5%) of the Monongahela River displayed the highest prevalence of 100% largemouth bass ancestry, while Kimsey Run Lake (2.9%), East Lynn Lake (3.1%), Sleepy Creek Lake (4.2%), Upper Mud Lake (7.1%), Parker Hollow Lake (8.3%), and Stonewall Jackson Lake (9.5%) displayed the lowest prevalence. The three Monongahela River pools, Warden Lake, and Sutton Lake all displayed a median of 100% largemouth bass ancestry. All pools of the Ohio River except Pike Island displayed an average largemouth bass ancestry above 95% with a median of 96.9% in each of the five pools. Pike Island only had a sample size of 3, resulting in a lower average and median ancestry, and displayed 0 individuals with 100% pure largemouth bass ancestry, which is likely an artifact of the small sample size.

### 3.3. ddRAD-Seq Ancestry Inferences, Population Structuring, and Diversity Evaluation

**Figure 2:**
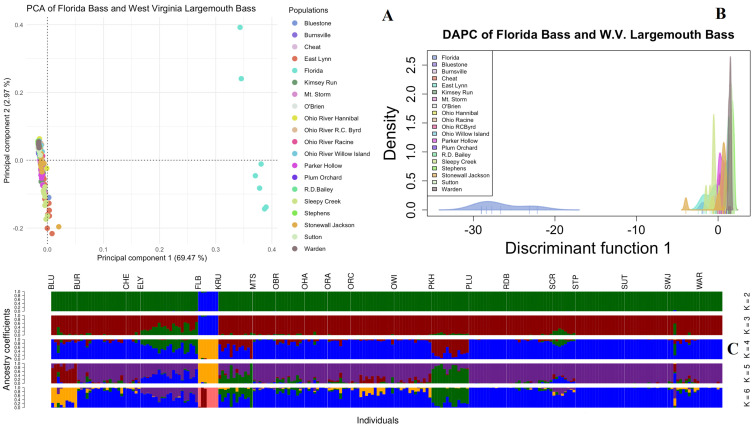
PCA (**A**), DAPC (**B**), and LEA ancestry assignment (**C**) plots of 226 largemouth bass from 19 sampling locations in West Virginia and 4 known Florida bass and 3 putative F1 hybrids as one population using 2772 SNPs. From left to right in section C, the populations are as follows: Bluestone (BLU), Burnsville (BUR), Cheat (CHE), East Lynn (ELY), Florida bass (FLB), Kimsey Run (KRU), Mt. Storm (MTS), O’Brien (OBR), Ohio River Hannibal Pool (OHA), Ohio River Racine Pool (ORA), Ohio River Willow Island Pool (OWI), Parker Hollow (PKH), Plum Orchard (PLU), R.D. Bailey (RDB), Sleepy Creek (SCR), Stephens (STP), Sutton (SUT), Stonewall Jackson (SWJ), and Warden (WAR).

The population structuring of Florida bass compared to West Virginia largemouth bass shows clear separation between the two species in all models and a lack of introgression between the two species within West Virginia populations. Initial population structuring inferences using PCA (Figure 2A) shows the clear separation of the four known Florida bass and three putative F1 hybrids on the first principal component, explaining 69.47% of total variance in the dataset. An optimal K of 2 was found using k-means clustering on a PCA transformation of the dataset (Supplementary Figure S1) and when K = 2 is used in the DAPC analysis, the Florida bass group again clusters together with all West Virginia largemouth bass form a second distinct cluster (Figure 2B). With LEA ancestry inference (Figure 2C), the Florida bass and F_1_ hybrid population is distinct from all West Virginia largemouth bass at K= 2 through K= 6. The cross-entropy validation of the model showed an equal likelihood of K = 4–6 (Supplementary Figure S2), with sub-structuring occurring in West Virginia largemouth bass at K = 3, 4, and 5. At K = 6, the two putative F_1_ hybrids from Millers Creek Reservoir, TX, cluster together as unique within the Florida bass population. At all K values, however, no ancestry assignment is observable for the Florida bass population in any sampled West Virginia largemouth bass. Pairwise *F*_ST_ comparisons showed that *F*_ST_ ranged from 0.563 to 0.705 between the Florida bass population and the 19 West Virginia largemouth bass populations. Among West Virginia populations, the *F*_ST_ values ranged from 0.016 to 0.189, with the highest pairwise *F*_ST_ being between Mt. Storm and Warden lakes (Supplementary Figure S3).

**Figure 3:**
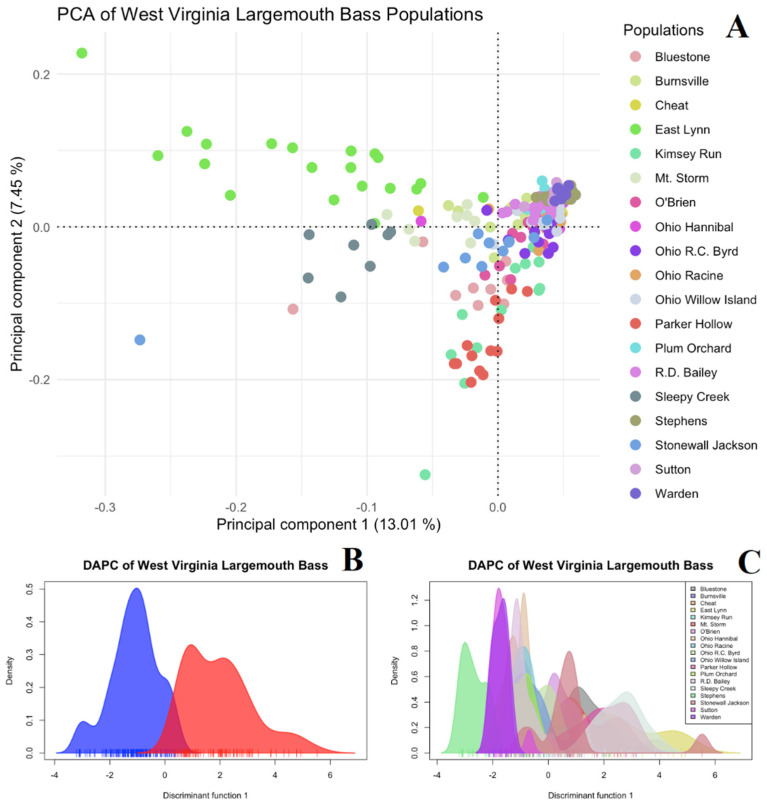
PCA (**A**) and DAPC plots by group, with each color (red and blue) representing the two genetic groups as defined by K = 2 using *k*-means clustering (**B**) and by population (**C**) for 226 largemouth bass from 19 sampling locations in West Virginia utilizing 2618 SNPs.

With the removal of Florida bass from the West Virginia largemouth bass dataset, the sub-structuring of West Virginia largemouth bass can be discerned. Initial population structuring inferences using PCA (Figure 3A) shows largemouth bass from East Lynn Lake Sleepy Creek Lake cluster apart from the main West Virginia cluster along the first principal component, which explains 13.01% of the total variance. Two individuals from Sutton Lake and one individual from Stonewall Jackson also cluster apart on the first principal component, with Mt. Storm being intermediary between the main cluster and the East Lynn and Sleepy Creek individuals.

**Figure 4:**
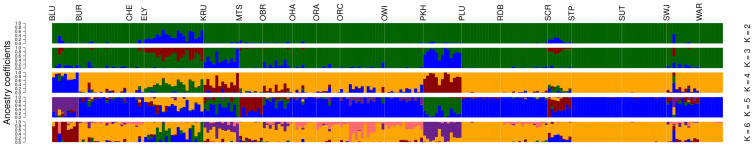
Ancestry plots generated by the *LEA* sparse nonnegative matrix factorization ancestry inference model for K = 2–6 among 226 largemouth bass from 19 sampling locations across West Virginia utilizing 2618 SNPs. From left to right, the populations are as follows: Bluestone (BLU), Burnsville (BUR), Cheat (CHE), East Lynn (ELY), Kimsey Run (KRU), Mt. Storm (MTS), O’Brien (OBR), Ohio River Hannibal Pool (OHA), Ohio River Racine Pool (ORA), Ohio River Willow Island Pool (OWI), Parker Hollow (PKH), Plum Orchard (PLU), R.D. Bailey (RDB), Sleepy Creek (SCR), Stephens (STP), Sutton (SUT), Stonewall Jackson (SWJ), and Warden (WAR).

K = 1 and 2 both showed similar likelihoods using the k-means clustering of the PCA-transformed data (Supplementary Figure S4), while LEA cross-entropy validation showed that K = 4 had the lowest cross-entropy criterion over fifty runs of K = 1–12 (Supplementary Figure S5). At K = 2, the DAPC plots show slight overlap between the two clusters (Figure 3B), with largemouth bass from Sleepy Creek, East Lynn, and Mt. Storm and one individual from Stonewall Jackson forming the second cluster apart from the main West Virgina cluster (Figure 3C). LEA ancestry assignment at K = 2 shows that largemouth bass from East Lynn and Sleepy Creek Lake show a unique ancestry, as well as one individual from Stonewall Jackson and Bluestone Lake (Figure 4). At K = 3, Parker Hollow and Kimsey Run Lake display a third ancestry most prominently, O’Brien Lake to a lesser extent, and minimally in Bluestone and Stonewall Jackson Lake individuals. At K =4, the best performing model, Parker Hollow and Kimsey Run Lake cluster together, East Lynn Lake highlights a unique ancestry that is also found but is much less prominent in Sleepy Creek Lake, Bluestone Lake shows a third unique ancestry that is also found to a lesser extent in R.C. Byrd and Willow Island pools of the Ohio River, and an over-arching Ohio River strain is found prominently in all other populations. Pairwise F_ST_ comparisons ranged from 0.001 to 0.191, with the highest F_ST_ being between Lake Stephens and Mt. Storm (Supplementary Figure S6). Overall, Ohio River pools displayed the lowest pairwise F_ST_ values throughout the state of West Virginia among each other, while Mt. Storm and Lake Stephens each displayed the highest F_ST_ values among all pairwise comparisons. When investigating pairwise F_ST_ values among populations that consist of only the Ohio River strain, values ranged from 0.004 to 0.191 with the Ohio River pool populations displaying the lowest F_ST_ among pairwise comparisons. Mt. Storm, Lake Stephens, and Warden Lake stood out as distinct among all other populations, and when these populations are not considered, F_ST_ values ranged from 0.004 to 0.094 with the highest difference being between Cheat and Sutton lakes (Supplementary Figure S7).

Genomic diversity was highest among sample locations that were genetically distinct, including Kimsey Run, Parker Hollow, Bluestone, and East Lynn Lake (Table 4). These sample locations had a higher π, Watterson’s Θ, and observed heterozygosity compared to populations largely consisting of largemouth bass of the dominant strain found throughout West Virginia such as Sutton, Warden, and Plum Orchard lakes and the various Ohio River pools. Among the four genetically distinct populations, Parker Hollow and East Lynn lakes had the highest effective population sizes, and East Lynn Lake had the highest number of private alleles among all sampled populations.

Among populations consisting of the dominant Ohio River strain found throughout the state, Plum Orchard, Warden, Mt. Storm, Sutton, and Lake Stephens had the lowest observed heterozygosity, while R.D. Bailey and O’Brien lakes had the highest. Warden, Plum Orchard, Warden, Sutton, and Lake Stephens also had the lowest π and Watterson’s Θ among all Ohio River strain populations with O’Brien and Mt. Storm lakes along with the R.C. Byrd and Willow Island pools of the Ohio River having the highest π and O’Brien Lake, R.C. Byrd, and Willow Island pools having the highest Watterson’s Θ. Warden, R.D. Bailey, Plum Orchard, and Lake Stephens had the highest effective population sizes, while Burnsville and the Ohio River pools had the lowest. F_IS_ was highest in Mt. Storm Lake, R.C. Byrd, and the Hannibal pools of the Ohio River, and lowest in Lake Stephens, Cheat, and R.D. Bailey lakes. The R.C. Byrd pool of the Ohio River, Mt. Storm, and Burnsville lakes had the highest number of private alleles among Ohio River strain populations, while Cheat Lake had zero private alleles. No SNPs were corroborated between the two models to detect outlier SNPs potentially undergoing selection, leading to no SNPs classifying as putatively under selection in West Virginia largemouth bass populations.

## 4. Discussion

Through the utilization of both GT-seq and dd-RAD sequencing approaches, the results of the present study show there is no introgression or successful introduction of Florida bass in West Virginia waters. The East Lynn and Sleepy Creek lakes showed the lowest percentage of pure largemouth bass in the NEWHYBRIDS genotype classification analysis, with both populations having less than 50% of sampled individuals classifying as pure largemouth bass. Despite these results, no largemouth bass from East Lynn or Sleepy Creek or any of the 226 sequenced largemouth bass from 19 sampling locations across the state of West Virginia showed Florida bass ancestry in the *LEA* ancestry assignment. The success of Florida bass stockings varies in the alterations in largemouth bass populations, with different levels of impact on native gene pools having been observed. Recent research has found that after stocking Florida bass over a 9-year time period, the introgression of Florida bass alleles was found in at least one individual at every sample site throughout the 16,000 ha reservoir, indicating the capability of population-wide introgression in a very large system [16]. Further inquiry into the same system revealed that despite an increase in Florida bass allele prevalence in the system, there was no contribution to greater growth potential, survival, or catchability in trophy-sized largemouth bass, highlighting that the shift in Florida bass allele prevalence is not sufficient for producing a trophy potential in the system [5]. Despite the observed ability of Florida bass to introgress into native largemouth bass populations, no introgression was found in the current study, indicating the lack of reproductive success of previously stocked Florida bass in West Virginia or a lack of introduction of Florida bass.

The high percentage of largemouth bass classifying as non-pure largemouth bass in the East Lynn and Sleepy Creek lakes can be explained by the actual SNP panel used in the GT-seq analysis. The 25 SNP panel utilized in the present study was validated against largemouth bass from Sugar Lake, Minnesota, and an unknown lake in Illinois, with both of these populations being fixed for the largemouth bass alleles in the previous study [12]. These populations likely have different allele frequencies within their native largemouth bass compared to those from West Virginia. Sugar Lake is located about 120 km south of the headwaters of the Mississippi River in Lake Itasca, while many of the populations sampled in the present study are located in the Ohio River, a tributary of the Mississippi River, and tributaries of the Ohio River [49]. While some individual samples contained 100% fixation for the largemouth bass allele across all sampled SNPs, no populations in the present study were completely fixed for the largemouth bass allele at all SNPs. This prevalence of Florida bass alleles in the dataset can be explained by recent research that shows the Florida bass has a much larger native range than previously described that extends to the Atlantic Coastal Plain, including Florida, Georgia, South Carolina, and North Carolina [3]. In the same study, the sub-structuring of largemouth bass into three genetic groups was also observed, where genetic groups were clustered together geographically and included a northcentral group throughout the Mississippi River basin and the Laurentian Great Lakes [3]. The introgression of Florida bass ancestry was also found in locations with known introductions, including rivers in Virginia that drain into the Chesapeake Bay. Our ddRAD-sequencing results found no Florida bass ancestry in any sequenced individuals, despite West Virginia sitting on the far eastern extent of the Mississippi River basin and being closer geographically to regions of the Florida bass native range than those of northern largemouth bass. Therefore, it can be deduced that the observed Florida bass alleles in the gene-linked SNPs utilized in the present study naturally occur in West Virginia largemouth bass populations and are not a result of Florida bass introgression or introductions. While these gene-linked SNPs may be fixed between northern largemouth bass populations and Florida bass, the Florida bass allele naturally occurs in West Virginia largemouth bass populations with low frequency owing to the potential distinctiveness of West Virginia largemouth bass compared to those in the northern Mississippi River Basin and the Laurentian Great Lakes [3,12]. The GT-Seq method employed in the current study is beneficial for genotyping known SNPs between the two species at a higher quantity of samples and ascertaining Florida bass ancestry in a more cost-effective method [12,26]. The utilization of the ddRAD-seq protocol enhances our ability to ascertain Florida bass ancestry by generating a dataset with over 170× more SNPs and to compare a subset of the genotyped West Virginia largemouth bass to known Florida bass and putative F_1_ hybrids. This combined approach lead us to the conclusion that there is no Florida bass ancestry in the state of West Virginia.

When investigating population structuring among West Virginia largemouth bass populations, four distinct genetic groups were identified. The dominant genetic group identified consisted of Ohio River populations and reservoirs in upstream tributaries, including Sutton, Burnsville, and Cheat lakes, and was found to some extent in every sampled population in the present study. Largemouth bass native to West Virginia would all be of this Ohio River strain origin, with the Ohio River watershed encompassing the vast majority of the state (excluding the eastern panhandle of the state that is a part of the Potomac River watershed [50]). Seeing as the Ohio River is the dominant watershed in the state of West Virginia, it is unsurprising that the Ohio River strain is the dominant strain found in largemouth bass populations throughout the state. A second cluster was found to be unique to Bluestone Lake, a dammed portion of the New River and its tributary the Bluestone River that represents the third largest body of water in the state. Largemouth bass are considered to have been introduced above Kanawha Falls, a natural barrier to fish passage upstream that is found downstream of the confluence of the Gauley and New rivers that form the Kanawha River [50,51]. This natural fish passage barrier has persisted for more than a million years and prohibits the upstream movement of fish native to the Ohio River watershed, including largemouth bass. The distinct genetic population found in Bluestone Lake is likely indicative of whatever source population was used to introduce largemouth bass into the system and could also be genetically similar to largemouth bass from upstream in the New River that includes Virginia and North Carolina. Bluestone Lake was of particular interest for potential Florida bass introgression due to the introduction of F1 Florida bass x largemouth bass hybrids into Claytor Lake, an upstream impoundment of the New River, by the Virginia Department of Wildlife Resources [52]. The potential movement of these F_1_ hybrids downstream into West Virginia waters, particularly Bluestone Lake and the New River, is of interest to management strategies. Current results, however, indicate no introgression of Florida bass ancestry in Bluestone Lake, highlighting that the unique genetic group of Bluestone Lake is of largemouth bass origin and not due to the introgression of F_1_ hybrids into the fishery. The lack of introgression of Florida bass alleles in Bluestone Lake also indicates several findings: there is limited to no gene flow of F_1_ hybrids from Claytor Lake into the system, any F_1_ hybrids that may have made their way into Bluestone Lake were simply not sampled, and any F_1_ hybrids that may have made their way into Bluestone Lake are not observably contributing to the genetic composition of Bluestone Lake. This genetically distinct ancestry was also found to a lesser extent in the R.C. Byrd pool of the Ohio River, where the Kanawha River drains into the Ohio River. This ancestry being found in the R.C. Byrd pool could be due to downstream movement of largemouth bass, or that this ancestry is unique to the Kanawha River and that Kanawha River largemouth bass were used in the introduction of largemouth bass into Bluestone Lake. The Kanawha River was not sequenced in the present study, leaving a gap of insight into the relationship between largemouth bass directly below Kanawha Falls and those in Bluestone Lake.

A third genetic group was identified in Kimsey Run and Parker Hollow lakes. These two lakes are in close geographic proximity to each other, separated by only 12 km, and are impoundments of the Kimsey Run and Parker Hollow creeks, respectively, with both creeks being tributaries to the Lost River, the headwaters of the Cacapon River. This unique ancestry was notably absent from Warden Lake, a nearby reservoir of Moores Run, a tributary of the Cacapon River. The Cacapon River is a third-order tributary of the Potomac River, where largemouth bass in West Virginia are designated as introduced and are not commonly found, with only 3 individuals having been previously sampled compared to 22 of the native smallmouth bass (*Micrpterus dolomieu*) [50,53]. The unique ancestry of the Kimsey Run and Parker Hollow lakes is likely due to the introduction and establishment of largemouth bass from a source that is not of Ohio River strain origin, like that of Warden Lake, which is dominated by Ohio River strain largemouth bass. The last genetically distinct ancestry was found predominantly in East Lynn Lake while being largely absent from other populations in the state. East Lynn Lake is situated in southwestern West Virginia and is an impoundment of the headwaters of East Fork Twelvepole Creek, a first-order tributary of the Ohio River by way of Twelvepole Creek. The East Lynn Lake population is an admixture of the Ohio River strain ancestry, and this observed unique but unknown ancestry is highly introgressed in the population. Largemouth bass from the nearby state of Kentucky may have been used to supplement the existing native largemouth bass populations, owing to the unique ancestry found predominantly in East Lynn Lake compared to any other sampled populations in West Virginia. This would also explain the high number of private alleles found in the East Lynn Lake population, where the introduction of new alleles from non-native largemouth bass would increase the number of private alleles compared to populations that did not receive stocking from this unknown genetically distinct population [24,54].

Aside from the unique ancestry found in East Lynn Lake, all other distinct genetic ancestries discovered in the present study are found predominantly in non-native introduced largemouth bass populations, including those at Bluestone, Parker Hollow, and Kimsey Run lakes [50]. Due to this observed trend, it is most likely that East Lynn Lake was supplementarily stocked with largemouth bass of non-Ohio River strain origin and that the Ohio River ancestry is the only genetically distinct native ancestry of largemouth bass in the state of West Virginia due to the lack of structuring occurring in any other native populations. Due to West Virginia’s geographical location on the eastern extent of the largemouth bass’ native non-introduced range, known sub-structuring of largemouth bass populations across its native range having been observed, and divergence in at least one other native Ohio River strain fish having been previously observed due to the introduction of a non-native genetic strain as a consequence of stocking, the introduction of Florida bass and/or the Florida bass x largemouth bass F_1_ hybrid into the state should be avoided to conserve native genetic diversity [3,55,56]. The introduction of Florida bass or the F_1_ hybrid into reservoirs that already contain non-native introduced largemouth bass populations could also lead to issues, given the possibility of fish movement by anglers, and it should likely be avoided to minimize these conservation concerns.

Genomic diversity metrics among West Virginia largemouth bass populations, including observed heterozygosity, Watterson’s Θ, and π, were highest in populations consisting of largemouth bass of non-Ohio River strain origin, including Bluestone, Kimsey Run, Parker Hollow, and East Lynn lakes. The observed heterozygosity was lower in all Ohio River strain origin populations when analyzed with these admixed populations, introducing a Wahlund effect of increased homozygosity due to sub-structuring within the dataset [57,58]. After the removal of these populations and admixed individuals, the genetic diversity of Ohio River strain largemouth bass populations could be inferred and was highlighted by populations from the Ohio River pools displaying some of the highest *F*_IS_ and lowest *N*_e_ values, while also having the highest π compared to inland reservoirs. This difference between the riverine populations and the inland reservoirs can potentially be explained by the reservoirs being able to sustain a higher census population and carrying capacity of largemouth bass compared to the Ohio River populations. Reservoirs create an artificial transitional ecosystem between a lake and river, where habitat generalists, such as the largemouth bass, tend to flourish [59]. The largemouth bass’ preference for habitats that have higher turbidity, vegetative pools, logs, and other habitat structures allows for them to flourish in these impounded systems [59,60,61]. An increase in the census population compared to the Ohio River populations would also increase the effective population size, with an increase in population size also allowing for a higher likelihood of the mating of unrelated individuals. The increased π in the Ohio River populations could be influenced by the observed ancestry of the genetically distinct Bluestone Lake ancestry in several of the Ohio River pools, notably the R.C. Byrd pool, which would also downwardly bias estimates of *N*_e_ [62]. This influence of the Bluestone Lake ancestry of unknown origin would allow for the introduction of new alleles that are absent from populations of pure Ohio River strain origin, with the R.C Byrd pool displaying the highest number of private alleles compared to any other population, with three times as many as the next highest Ohio River pool. Further research into the native Kanawha River and introduced New River largemouth bass populations is needed to elucidate if this ancestry increases in prevalence in more upstream populations and to identify the most likely source of this unknown genetically distinct ancestry [50].

## 5. Conclusions

The results of the present study can be utilized to conserve native largemouth bass populations and inform future management directions. With a lack of observed introgression or presence of Florida bass ancestry in West Virginia, the stocking of Florida bass or the F_1_ hybrid should be avoided in the Ohio River watershed to conserve native genetic diversity. Additionally, the stocking of Florida bass or the F_1_ hybrid should likely be avoided in other watersheds of the state to prevent the movement of this species and/or its hybrids into other waters by anglers. With a noted lack of sub-structuring in native Ohio River strain largemouth bass populations, future supplementary stocking of largemouth should prioritize the stocking of the Ohio River strain ancestry throughout the state. Ohio River pools’ largemouth bass showed higher genetic diversity but reduced effective population sizes compared to inland reservoirs, indicating the need to potentially alleviate this observed decreased *N*_e_ through the stocking of unrelated largemouth bass into Ohio River pools. With low differentiation among pools, the propagated spawning of individuals from different pools would assist in reducing the observed higher inbreeding in Ohio River populations compared to the inland reservoirs.

## Figures and Tables

**Table 1:** The diagnostic SNP used to ascertain Florida bass allele prevalence in West Virginia is presented, along with the position of the SNP in the read, the reads generated, and the primers used for each locus. The position of the variant site is presented in the parenthesis in the locus column. The forward primer is presented first (F), with the reverse primer (R) presented after the forward primer in the primer column. The variant SNP is presented in parentheses in the read column, with the largemouth bass allele presented first and the Florida bass allele presented second.

Locus	Primer	Read
8751 (22)	F: CGACAGGTTCAGAGTTCTACAGTCCGACGATCTTCTCTGGTGATCTGTGTGG R: GTGACTGGAGTTCAGACGTGTGCTCTTCCGATCTAGTGCTGTTCTTAGCCGTTC	CGGACACTCGCATGACTTGAG (A/G) CGCAGGGCTGGTGTTGTATGAACGG
11367-1 (10)	F: CGACAGGTTCAGAGTTCTACAGTCCGACGATCTCCCAGGATTTGCCTATCAG R: GTGACTGGAGTTCAGACGTGTGCTCTTCCGATCTGGAGGAGATTGTTGTGTGAG	AGAGGACAA (G/A) GACAAGAACATCCTTATCCCCGGCCTCACTCA
6106 (19)	F: CGACAGGTTCAGAGTTCTACAGTCCGACGATCGGAGACTTTGATGTCAGAGG R: GTGACTGGAGTTCAGACGTGTGCTCTTCCGATCTTCCAAGACTGACTGAGTCAC	TCTGAGTGTTTGGGGAGA (C/T) CTCCTGTCAGCATTACACAAGTGA
2930 (11)	F: CGACAGGTTCAGAGTTCTACAGTCCGACGATCTCATTGTCCCTGACATGTGG R: GTGACTGGAGTTCAGACGTGTGCTCTTCCGATCTGGCTGTGAAAAAGCTAGACC	CTGAAGGACG (A/T) GTATCTGCATACTGCAGCAAGCAAGTTCAATTTCCAT
33087 (24)	F: CGACAGGTTCAGAGTTCTACAGTCCGACGATCATGGTGACAGCCACAGCATC R: GTGACTGGAGTTCAGACGTGTGCTCTTCCGATCTTGTGAAGTTCGGAGCTGATG	AGCCAGCAGGTCCACCCCCTGCA (G/A) CATGAGGGCACGAGCATCAGCTCCG
32455 (42)	F: CGACAGGTTCAGAGTTCTACAGTCCGACGATCGAGATCATTCGTCACTCCTG R: GTGACTGGAGTTCAGACGTGTGCTCTTCCGATCTAATGTTGACCACTGGTAGAG	GGATTTAATTAGGGATTTCTAGTGAGCATAAATGTCACTTT (A/C) ATTTGTGTTAATTAACTCTACCAGTGGTCAACATTAGATCGGAAGAGCAC
11367 (33)	F: CGACAGGTTCAGAGTTCTACAGTCCGACGATCCAATGTTGGGCAGTTCAGAG R: GTGACTGGAGTTCAGACGTGTGCTCTTCCGATCTTCATCCTTAGCTTTGACCAG	CAGCCAGGCGTGATCCCCCGGGCTGTCCGTGA (A/G) GTCTTCAATCTGGTCAAAGCTAAGGA
17385 (34)	F: CGACAGGTTCAGAGTTCTACAGTCCGACGATCCACTGCTAATGGAGTAGCTG R: GTGACTGGAGTTCAGACGTGTGCTCTTCCGATCTCAGCAGTGGCTTGGAAATAC	AAAAGTAAAAATGTATTAAAGAGGAAAAACAGC (G/A) TTGTAGTCTAGTATTTCTAAGCCACT
35139 (33)	F: CGACAGGTTCAGAGTTCTACAGTCCGACGATCTCTCACGTCTGTATGTGCTG R: GTGACTGGAGTTCAGACGTGTGCTCTTCCGATCTTTTGTTGACCCCGTGGTAAC	TTTAGGGTCAAAACCATACAGGAGAATGAAGG (C/T) GCTTAAAAGTTACCAC
5903 (51)	F: CGACAGGTTCAGAGTTCTACAGTCCGACGATCCGTTCTAGTTCTTATGTCAC R: GTGACTGGAGTTCAGACGTGTGCTCTTCCGATCTTCCCTCCTAATCTGAACCTG	TCTTTGTTATTTTGGTATCACTGTTAAATCAAAGGGAAAGAGGATTGTC (A/T) TCACTTGCAGGTTCAGATTAGGAGGGAAGATCGGAAGAGCACACGTCTGAACT
31992 (34)	F: CGACAGGTTCAGAGTTCTACAGTCCGACGATCTTTAGTTCCACTGCCACCAC R: GTGACTGGAGTTCAGACGTGTGCTCTTCCGATCTCTGGTGGATCTGGATCCAGT	CAGATACACCAATGACAAATGATCCAGGAAAAC (T/C) GGCGTCAGGGTAGGCAAA
15421 (9)	F: CGACAGGTTCAGAGTTCTACAGTCCGACGATCCATCTACTACACCCACACTG R: GTGACTGGAGTTCAGACGTGTGCTCTTCCGATCTTGCAGAGGAGCATGTTGATG	ATGGTTGC (A/T) CTGAGAACAAGGTCTTCATCAGCATCAACATGCTCCTCTGCAAG
2993 (13)	F: CGACAGGTTCAGAGTTCTACAGTCCGACGATCAAGACATAACGTGGTGCAAG R: GTGACTGGAGTTCAGACGTGTGCTCTTCCGATCTCTCCCACAGTATGTCAACTC	TTTTTTAGGTTC (A/T) CCTGGGACAGCATGTCTCTATAGAGCAAGAGTTGACATAC
19092 (12)	F: CGACAGGTTCAGAGTTCTACAGTCCGACGATCGCAAAAATGTGTTAAACGGG R: GTGACTGGAGTTCAGACGTGTGCTCTTCCGATCTGCTACTAGGCTGAAACCTAC	TTATTTAGAAT (T/G) AGTAGGTCAGCAACTATGTCTGCATTGCAGTTTTCAGTAGGT
31857 (9)	F: CGACAGGTTCAGAGTTCTACAGTCCGACGATCACTAAAACCACTGGCCAGTC R: GTGACTGGAGTTCAGACGTGTGCTCTTCCGATCTGGGAAAGTGGCCTCTAAAAG	CAGTCTTA (T/C) AGCACGCAGGGAGGGTTTTATCCAAAACCACAAAATAAATGA
26936 (15) *	F: CGACAGGTTCAGAGTTCTACAGTCCGACGATCTGCTCATGGTAAGCGTCCTC R: CGACAGGTTCAGAGTTCTACAGTCCGACGATCTGCTCATGGTAAGCGTCCTC	CATCTCACTCTCCA (A/G) CTTCTCACGAGCCTCACGCATGTTCTTCTCTACCTGTTG

An asterisk in the loci denotes that an observed diagnostic SNP between Florida bass and largemouth bass had a different base pair variation than previously found [12], but was found to be fixed during validation in the present study.

**Table 2:** Genotyping statistics of the 16 diagnostic SNPs that were used to assess Florida bass allele prevalence in West Virginia.

SNP ID	Average Read Depth	Genotype Rate	Florida Bass Allele Frequency	Largemouth Bass Allele Frequency
17385	2166.72	1.00	0.03	0.97
32455	2485.42	1.00	0.11	0.89
31992	3015.77	1.00	0.12	0.88
2930	3187.93	1.00	0.05	0.95
2993	3439.82	1.00	0.08	0.92
5903	3607.41	1.00	0.03	0.97
19092	3712.35	1.00	0.06	0.94
6106	4120.89	1.00	0.04	0.96
8751	5388.11	1.00	0.24	0.76
11367-1	5390.22	1.00	0.04	0.96
31857	5504.14	1.00	0.05	0.95
35139	5838.82	1.00	0.06	0.94
33087	7291.91	1.00	0.05	0.95
11367	8838.12	1.00	0.08	0.92
15421	9305.76	1.00	0.04	0.96
26936	9512.58	1.00	0.10	0.90

**Table 3:** The Florida bass allele prevalence for each sampling location is presented, with the sample size for each sampling location found in the parenthesis. The number of individuals with an above 80% probability of being a pure largemouth bass is found in the second column, with the total percentage of individuals by sample location presented in the parenthesis. The second most prevalent genotype assignment is presented in parentheses with the number of individuals displaying an 80% or higher probability of belonging to that category. The average, median, and range of largemouth bass ancestry is presented in the final three columns.

Population (*n*)	Pure LMB (% of Population)	Second Most Prevalent Assignment	Average Ancestry	Median Ancestry	Range Ancestry
Beech Fork Lake (30)	30 (100%)	N/A	93.1%	93.8%	87.5–100%
Bluestone Lake (19)	14 (73.7%)	LMB_Bx (5)	95.4%	96.9%	71.9–100%
Burnsville Lake (19)	19 (100%)	N/A	96.5%	96.9%	90.6–100%
Cheat Lake (18)	17 (94.4%)	LMB_Bx (1)	93.1%	93.8%	87.5–100%
East Lynn Lake (32)	15 (46.9%)	LMB_Bx (13)	86.5%	87.5%	56.3–100%
Elk Fork Lake (32)	24 (75.0%)	LMB_Bx (2)	94.9%	96.9%	84.4–100%
Kanawha River Winfield Pool (14)	13 (92.9%)	LMB_Bx (1)	97.3%	96.9%	90.6–100%
Kimsey Run Lake (35)	30 (85.7%)	LMB_Bx (2)	93.4%	93.8%	84.4–100%
Monongahela River Morgantown Pool (22)	19 (86.4%)	LMB_Bx (2)	95.9%	100.0%	81.3–100%
Monongahela River Opekiska Pool (29)	24 (82.8%)	LMB_Bx (5)	95.4%	100.0%	75.0–100%
Monongahela River Point Marion Pool (47)	43 (91.5%)	LMB_Bx (4)	97.0%	100.0%	78.1–100%
Mount Storm (38)	38 (100%)	N/A	96.1%	96.9%	87.5–100%
North Bend Lake	28 (93.3%)	N/A	96.3%	96.9%	87.5–100%
O’Brien Lake (38)	38 (100%)	N/A	97.0%	96.9%	87.5–100%
Ohio River Belleville Pool (44)	42 (95.5%)	LMB_Bx (1)	96.7%	96.9%	81.3–100%
Ohio River Hannibal Pool (21)	20 (95.2%)	LMB_Bx (1)	95.5%	96.9%	78.1–100%
Ohio River Racine Pool (23)	20 (87.0%)	LMB_Bx (1)	95.1%	96.9%	81.3–100%
Ohio River R.C. Byrd Pool (21)	20 (95.2%)	LMB_Bx (1)	96.9%	96.9%	87.5–100%
Ohio River Willow Island Pool (36)	34 (94.4%)	LMB_Bx (1)	96.6%	96.9%	81.3–100%
Ohio River Pike Island Pool (3)	3 (100%)	N/A	93.8%	93.8%	90.6–96.9%
Parker Hollow Lake (36)	33 (91.7%)	LMB_Bx (1)	92.4%	92.2%	81.3–100%
Plum Orchard Lake (20)	20 (100%)	N/A	97.2%	96.9%	81.3–100%
R.D Bailey Lake (22)	21 (95.5%)	N/A	95.0%	96.9%	84.4–100%
Sleepy Creek Lake (24)	10 (41.7%)	LMB_Bx (8)	84.8%	87.5%	62.5–100%
South Mill Creek Lake (36)	32 (88.9%)	LMB_Bx (1)	92.8%	93.8%	84.4–100%
Stephens Lake (34)	34 (100%)	N/A	97.9%	96.9%	90.6–100%
Stonewall Jackson Lake (21)	17 (80.9%)	LMB_Bx (3)	91.2%	90.6%	65.6–100%
Sutton Lake (23)	23 (100%)	N/A	98.8%	100.0%	93.8–100%
Tygart Lake (20)	18 (90%)	LMB_Bx (1)	95.9%	96.9%	71.9–100%
Upper Mud Lake (28)	22 (78.6%)	LMB_Bx (6)	90.3%	90.6%	71.9–100%
Warden Lake (41)	41 (100%)	N/A	98.3%	100.0%	93.8–100%
Total (856)	760 (88.8%)	LMB_Bx (62)	94.9%	96.9%	56.3–100%

**Table 4:** Estimates of genetic diversity for West Virginia largemouth bass from 19 sampling locations using 2618 SNPs in the first row, and 3599 SNPs in the second row for populations and individuals of the dominant Ohio River West Virginia strain, when applicable. The sample size for each analysis is in the parenthesis. The mean frequency of the most frequent allele at each locus (*P*), percentage of polymorphic loci (*P* loci), number of private alleles (Private), nucleotide diversity (π; standard error SE), observed heterozygosity (*H*_O_), expected heterozygosity (*H*_E_), number and percentage of SNPs found to be out of Hardy–Weinberg equilibrium (HWE), inbreeding coefficient (*F*_IS_), Watterson’s Ɵ (Ɵ), Tajima’s D statistic (Tajima), and estimated effective population size (*N*_e_), with corresponding 95% confidence intervals, are presented.

Population	*P*	*P* loci	Private	π (SE)	*H*_O_ (SE)	*H*_E_ (SE)	HWE	*F*_IS_ (SE)	Ɵ	Tajima	*N*_e_ (95% CI)
Bluestone Lake (9)	0.922	0.119	16	0.123 (0.003)	0.099 (0.003)	0.116 (0.003)	28 (1.07%)	0.070 (0.009)	0.147	−0.028	10.1 (9.8–10.4)
Burnsville Lake (17)	0.935	0.120	5	0.100 (0.003)	0.082 (0.003)	0.097 (0.003)	108 (4.13%)	0.067 (0.015)	0.124	−0.027	46.3 (43.7–49.2)
	0.909	0.147	49	0.137 (0.003)	0.112 (0.002)	0.133 (0.003)	198 (5.50%)	0.089 (0.014)	0.158	−0.023	57.2 (55.0–59.5)
Cheat Lake (5)	0.941	0.069	0	0.093 (0.003)	0.076 (0.003)	0.083 (0.003)	7 (0.27%)	0.039 (0.007)	0.106	−0.017	∞
	0.919	0.091	0	0.127 (0.003)	0.103 (0.003)	0.114 (0.003)	16 (0.44%)	0.054 (0.006)	0.143	−0.024	∞
East Lynn Lake (20)	0.903	0.170	78	0.152 (0.003)	0.132 (0.003)	0.148 (0.003)	113 (4.31%)	0.077 (0.015)	0.165	−0.017	70.4 (67.5–73.5)
Kimsey Run Lake (12)	0.926	0.117	3	0.116 (0.003)	0.092 (0.003)	0.110 (0.003)	90 (3.44%)	0.078 (0.014)	0.138	−0.027	46.0 (42.1–50.7)
Lake Stephens (17)	0.954	0.068	4	0.067 (0.003)	0.060 (0.003)	0.065 (0.003)	44 (1.68%)	0.025 (0.012)	0.069	−0.003	81.0 (69.4–96.9)
	0.934	0.090	16	0.096 (0.003)	0.084 (0.003)	0.093 (0.003)	92 (2.56%)	0.038 (0.011)	0.094	−0.001	131.7 (115.9–152.4)
Mt. Storm Lake (8)	0.932	0.081	1	0.103 (0.003)	0.056 (0.002)	0.096 (0.003)	67 (2.56%)	0.124 (0.010)	0.114	−0.019	∞
	0.904	0.107	43	0.144 (0.003)	0.079 (0.002)	0.135 (0.003)	141 (3.92%)	0.167 (0.014)	0.155	−0.023	∞
O’Brien Lake (10)	0.934	0.101	3	0.103 (0.003)	0.083 (0.003)	0.097 (0.003)	32 (1.22%)	0.058 (0.013)	0.124	−0.025	∞
	0.909	0.130	14	0.140 (0.003)	0.113 (0.003)	0.133 (0.003)	58 (1.61%)	0.079 (0.012)	0.169	−0.034	∞
Ohio River Hannibal Pool (8)	0.938	0.091	0	0.098 (0.003)	0.066 (0.002)	0.091 (0.003)	28 (1.07%)	0.093 (0.010)	0.123	−0.031	92.8 (71.8–130.2)
	0.913	0.114	1	0.135 (0.003)	0.089 (0.002)	0.126 (0.003)	70 (1.95%)	0.126 (0.008)	0.164	−0.037	771.8 (331.3–∞)
Ohio River Racine Pool (8)	0.937	0.092	1	0.099 (0.003)	0.074 (0.003)	0.092 (0.003)	19 (0.73%)	0.068 (0.009)	0.117	−0.023	∞
	0.911	0.117	2	0.139 (0.003)	0.102 (0.003)	0.130 (0.003)	52 (1.17%)	0.099 (0.008)	0.157	−0.025	∞
Ohio River R.C. Byrd Pool (15)	0.932	0.114	21	0.105 (0.003)	0.077 (0.002)	0.101 (0.003)	138 (5.27%)	0.097 (0.016)	0.126	−0.025	30.5 (29.0–32.2)
	0.904	0.145	51	0.146 (0.003)	0.107 (0.002)	0.141 (0.003)	265 (7.36%)	0.133 (0.015)	0.168	−0.028	35.9 (34.8–37.1)
Ohio River Willow Island Pool (13)	0.934	0.109	4	0.102 (0.003)	0.081 (0.003)	0.098 (0.003)	113 (4.32%)	0.074 (0.012)	0.121	−0.022	41.4 (38.2–45.1)
	0.905	0.141	15	0.145 (0.003)	0.112 (0.002)	0.139 (0.003)	230 (6.39%)	0.109 (0.008)	0.165	−0.024	42.5 (40.7–44.5)
Parker Hollow Lake (13)	0.922	0.114	3	0.119 (0.003)	0.105 (0.003)	0.114 (0.003)	63 (2.41%)	0.044 (0.011)	0.124	−0.008	98.6 (85.4–116.5)
Plum Orchard Lake (13)	0.947	0.075	3	0.079 (0.003)	0.049 (0.002)	0.076 (0.003)	150 (5.73%)	0.093 (0.015)	0.093	−0.017	132.8 (96.3–211.4)
	0.925	0.098	9	0.111 (0.003)	0.069 (0.002)	0.106 (0.003)	260 (7.22%)	0.125 (0.13)	0.126	−0.020	134.1 (111.7–167.2)
R.D. Bailey Lake (16)	0.939	0.109	11	0.094 (0.003)	0.083 (0.003)	0.091 (0.003)	70 (2.67%)	0.042 (0.013)	0.113	−0.021	131.8 (111.2–161.2)
	0.913	0.140	30	0.132 (0.003)	0.116 (0.003)	0.128 (0.003)	133 (3.70%)	0.058 (0.012)	0.150	−0.020	153.7 (138.1–173.0)
Sleepy Creek Lake (8)	0.911	0.116	4	0.139 (0.003)	0.088 (0.003)	0.130 (0.003)	57 (2.18%)	0.139 (0.010)	0.161	−0.031	∞
Stonewall Jackson Lake (11)	0.923	0.129	4	0.122 (0.003)	0.074 (0.002)	0.116 (0.003)	162 (6.19%)	0.159 (0.015)	0.165	−0.050	32.8 (30.8–35.1)
Sutton Lake (15)	0.951	0.074	2	0.073 (0.003)	0.060 (0.002)	0.071 (0.003)	65 (2.48%)	0.042 (0.015)	0.081	−0.009	65.8 (57.2–77.3)
	0.930	0.097	7	0.104 (0.003)	0.086 (0.002)	0.100 (0.003)	135 (3.79%)	0.061 (0.014)	0.111	−0.009	72.5 (66.7–79.2)
Warden Lake (8)	0.954	0.049	2	0.068 (0.003)	0.050 (0.002)	0.063 (0.003)	24 (1.11%)	0.044 (0.009)	0.064	0.001	105.2 (67.0–265.5)
	0.935	0.066	8	0.095 (0.003)	0.070 (0.002)	0.089 (0.003)	52 (1.44%)	0.059 (0.008)	0.088	0.002	280.3 (156.6–1257.6)

## Data Availability

All datasets used in the current manuscript can be found online at Figshare at the following link: https://figshare.com/projects/Largemouth_Bass_West_Virginia/240881.

## Supplementary Materials

The following supporting information can be downloaded at https://www.mdpi.com/article/10.3390/biology14040392/s1. Figure S1. Optimal number of clusters using *k-means* clustering on the PCA transformed data on all sampled largemouth bass, Florida bass, and putative F_1_ hybrids using a total of 2772 SNPs. Figure S2. Optimal number of clusters inferred from *LEA* cross-entropy criterion following 50 runs of K 1–12 for all sampled largemouth bass, Florida bass, and putative F_1_ hybrids using a total of 2772 SNPs. Figure S3. Pairwise *F*_ST_ values between all sampled West Virginia largemouth bass populations and a population consisting of known Florida bass and putative F_1_ hybrids using a total of 2772 SNPs. Figure S4. Optimal number of clusters using *k-means* clustering on the PCA transformed data on all sampled West Virginia largemouth bass populations using a total of 2618 SNPs. Figure S5. Optimal number of clusters using *LEA* cross-entropy criterion averaged over 50 runs of K 1–12 on all sampled West Virginia largemouth bass populations using a total of 2618 SNPs. Figure S6. Pairwise *F*_ST_ values between all sampled West Virginia largemouth bass populations using a total of 2618 SNPs. Figure S7. Pairwise *F*_ST_ values between West Virginia largemouth bass populations dominated by the Ohio River strain ancestry using a total of 3599 SNPs.
