# Supplementary material for: Fishing for Florida Bass in West Virginia: Genomic Evaluation of Florida Bass Presence and Establishing Baselines of Genetic Structure and Diversity for Native Largemouth Bass
Source: Biology (Basel). 2025 Apr 9;14(4):392. doi: 10.3390/biology14040392 (PMC12024669; doi:10.3390/biology14040392)
Supplement: Supplementary file 1 [file biology-14-00392-s001.zip › biology-3553723-supplementary.pdf]

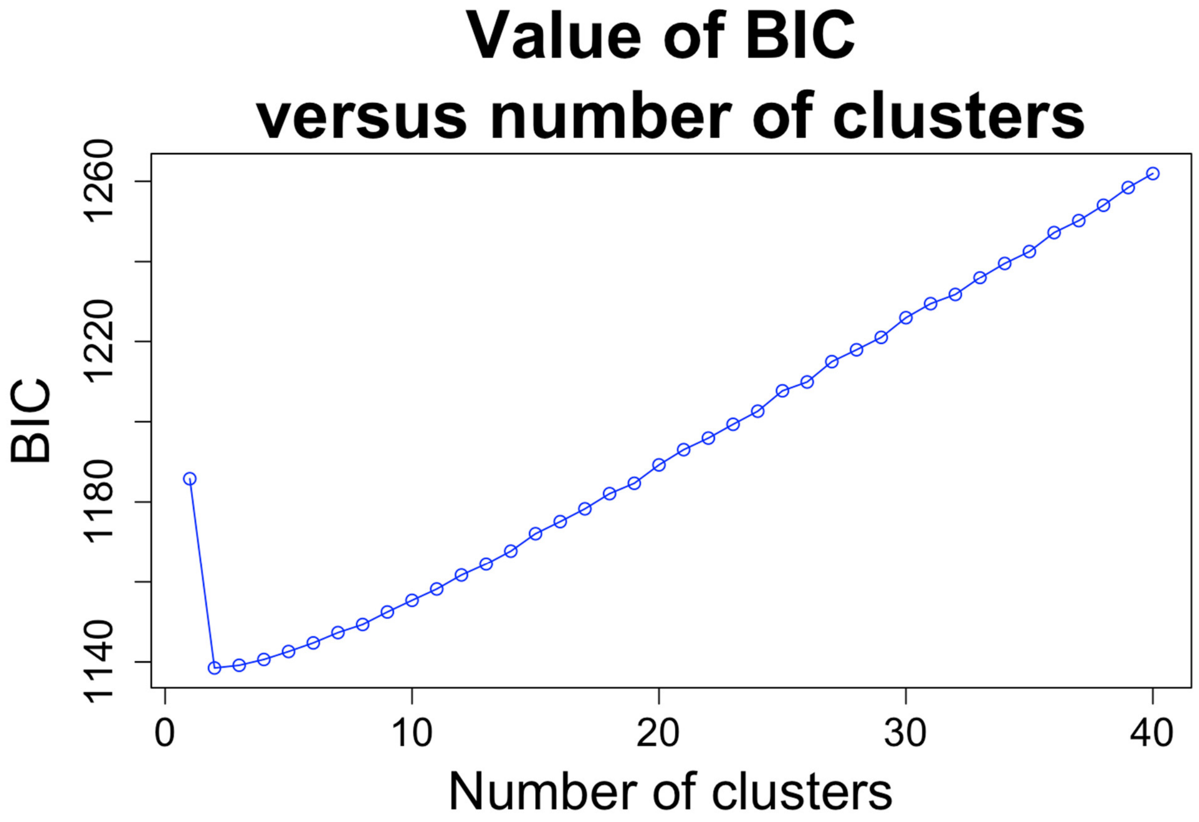


**Figure 1.** Optimal number of clusters using *k-means* clustering on the PCA transformed data on all sampled largemouth bass, Florida bass, and putative $F_1$ hybrids using a total of 2,772 SNPs.

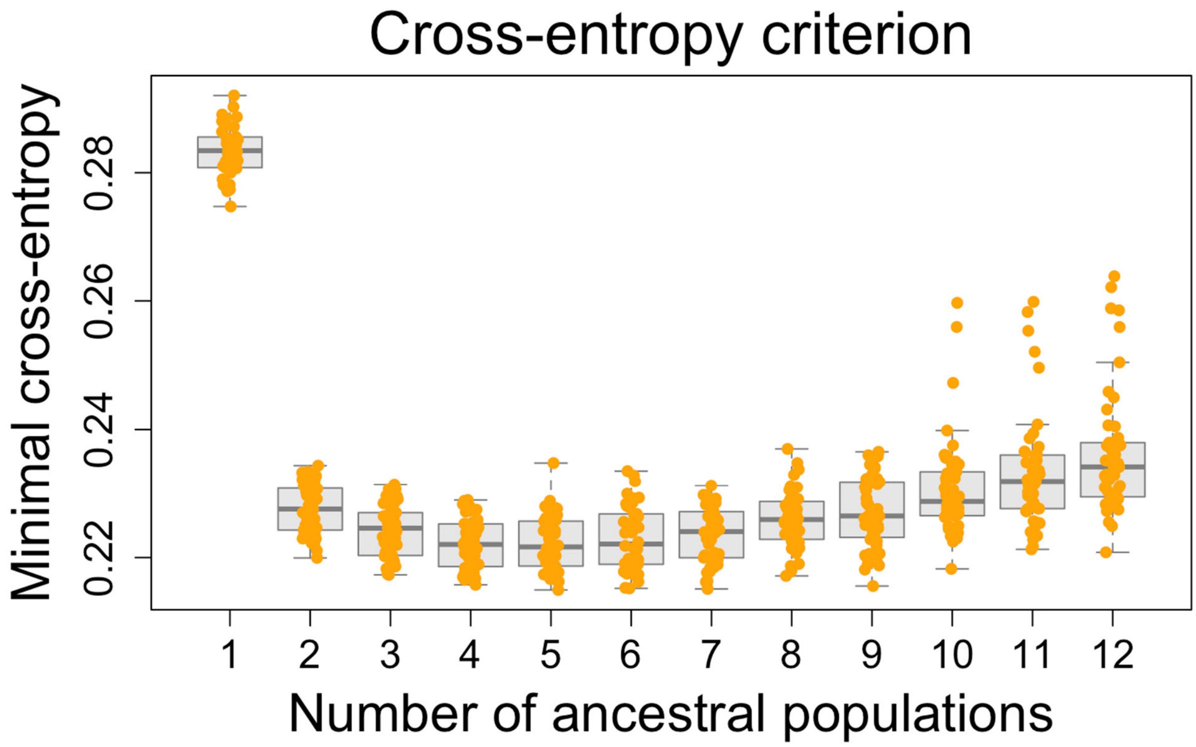


**Figure 2.** Optimal number of clusters inferred from *LEA* cross-entropy criterion following 50 runs of K 1-12 for all sampled largemouth bass, Florida bass, and putative $F_1$ hybrids using a total of 2,772 SNPs.

Weir & Cockerham 1984 Fst

| | BLUE | BURN | CHEAT | ELYN | FLB | KRUN | MTST | OBRN | OHHA | OHRA | OHRC | OHWI | PKHO | PLUM | RDBA | SCRK | STPH | SUTN | SWJ | WARD |
|---|---|---|---|---|---|---|---|---|---|---|---|---|---|---|---|---|---|---|---|---|
| BLUE | | 0.102 | 0.088 | 0.082 | 0.596 | 0.084 | 0.129 | 0.096 | 0.083 | 0.072 | 0.08 | 0.074 | 0.104 | 0.129 | 0.104 | 0.101 | 0.169 | 0.136 | 0.058 | 0.153 |
| BURN | | | 0.064 | 0.064 | 0.661 | 0.055 | 0.116 | 0.055 | 0.037 | 0.043 | 0.052 | 0.045 | 0.086 | 0.068 | 0.053 | 0.097 | 0.101 | 0.062 | 0.05 | 0.12 |
| CHEAT | | | | 0.055 | 0.591 | 0.065 | 0.11 | 0.057 | 0.016 | 0.041 | 0.04 | 0.032 | 0.088 | 0.076 | 0.061 | 0.084 | 0.129 | 0.082 | 0.021 | 0.138 |
| ELYN | | | | | 0.603 | 0.058 | 0.081 | 0.052 | 0.037 | 0.047 | 0.055 | 0.048 | 0.074 | 0.059 | 0.056 | 0.05 | 0.114 | 0.067 | 0.04 | 0.094 |
| FLB | | | | | | 0.619 | 0.605 | 0.622 | 0.609 | 0.61 | 0.646 | 0.64 | 0.625 | 0.664 | 0.667 | 0.563 | 0.705 | 0.684 | 0.599 | 0.64 |
| KRUN | | | | | | | 0.104 | 0.044 | 0.036 | 0.037 | 0.048 | 0.043 | 0.022 | 0.076 | 0.053 | 0.079 | 0.128 | 0.08 | 0.026 | 0.119 |
| MTST | | | | | | | | 0.1 | 0.088 | 0.102 | 0.114 | 0.109 | 0.123 | 0.124 | 0.12 | 0.103 | 0.18 | 0.13 | 0.077 | 0.189 |
| OBRN | | | | | | | | | 0.032 | 0.037 | 0.044 | 0.039 | 0.057 | 0.065 | 0.055 | 0.084 | 0.127 | 0.067 | 0.035 | 0.112 |
| OHHA | | | | | | | | | | 0.001 | 0.011 | 0.005 | 0.061 | 0.046 | 0.032 | 0.071 | 0.106 | 0.055 | 0.014 | 0.089 |
| OHRA | | | | | | | | | | | 0.007 | 0.008 | 0.055 | 0.058 | 0.039 | 0.078 | 0.117 | 0.062 | 0.02 | 0.08 |
| OHRC | | | | | | | | | | | | 0.018 | 0.068 | 0.063 | 0.044 | 0.091 | 0.103 | 0.064 | 0.029 | 0.083 |
| OHWI | | | | | | | | | | | | | 0.07 | 0.058 | 0.03 | 0.086 | 0.101 | 0.059 | 0.026 | 0.087 |
| PKHO | | | | | | | | | | | | | | 0.102 | 0.078 | 0.096 | 0.148 | 0.101 | 0.047 | 0.147 |
| PLUM | | | | | | | | | | | | | | | 0.059 | 0.107 | 0.133 | 0.043 | 0.053 | 0.132 |
| RDBA | | | | | | | | | | | | | | | | 0.094 | 0.091 | 0.049 | 0.043 | 0.096 |
| SCRK | | | | | | | | | | | | | | | | | 0.153 | 0.111 | 0.052 | 0.128 |
| STPH | | | | | | | | | | | | | | | | | | 0.1 | 0.098 | 0.182 |
| SUTN | | | | | | | | | | | | | | | | | | | 0.059 | 0.122 |
| SWJ | | | | | | | | | | | | | | | | | | | | 0.094 |
| WARD | | | | | | | | | | | | | | | | | | | | |

**Figure 3.** Pairwise $F_{ST}$ values between all sampled West Virginia largemouth bass populations and a population consisting of known Florida bass and putative $F_1$ hybrids using a total of 2,772 SNPs.

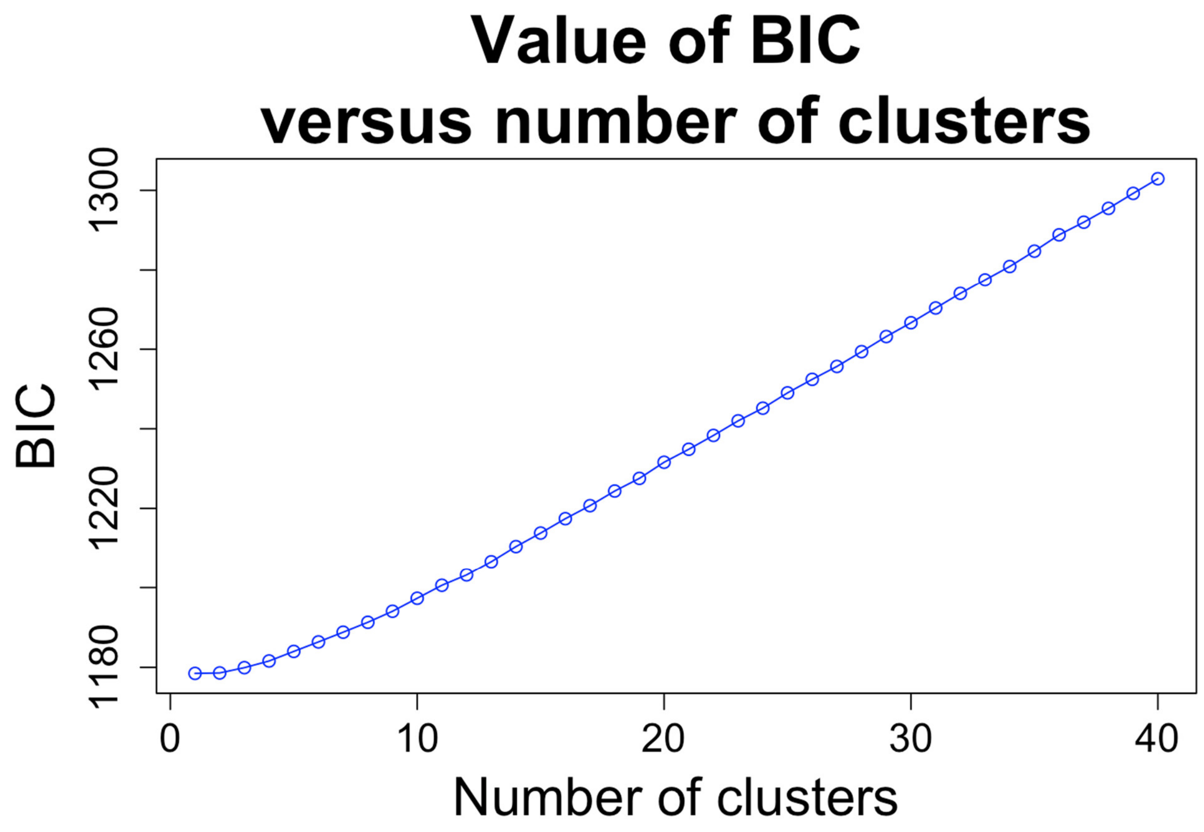


**Figure 4.** Optimal number of clusters using *k-means* clustering on the PCA transformed data on all sampled West Virginia largemouth bass populations using a total of 2,618 SNPs.

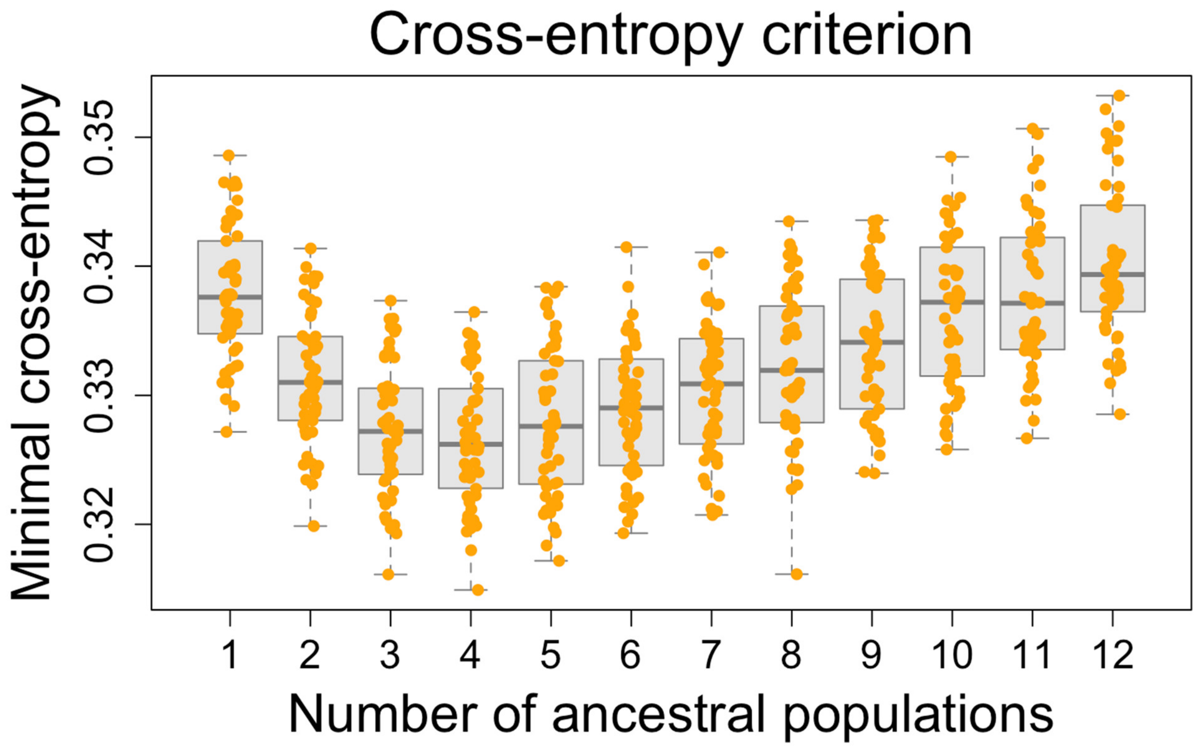


**Figure 5.** Optimal number of clusters using *LEA* cross-entropy criterion averaged over 50 runs of K 1-12 on all sampled West Virginia largemouth bass populations using a total of 2,618 SNPs

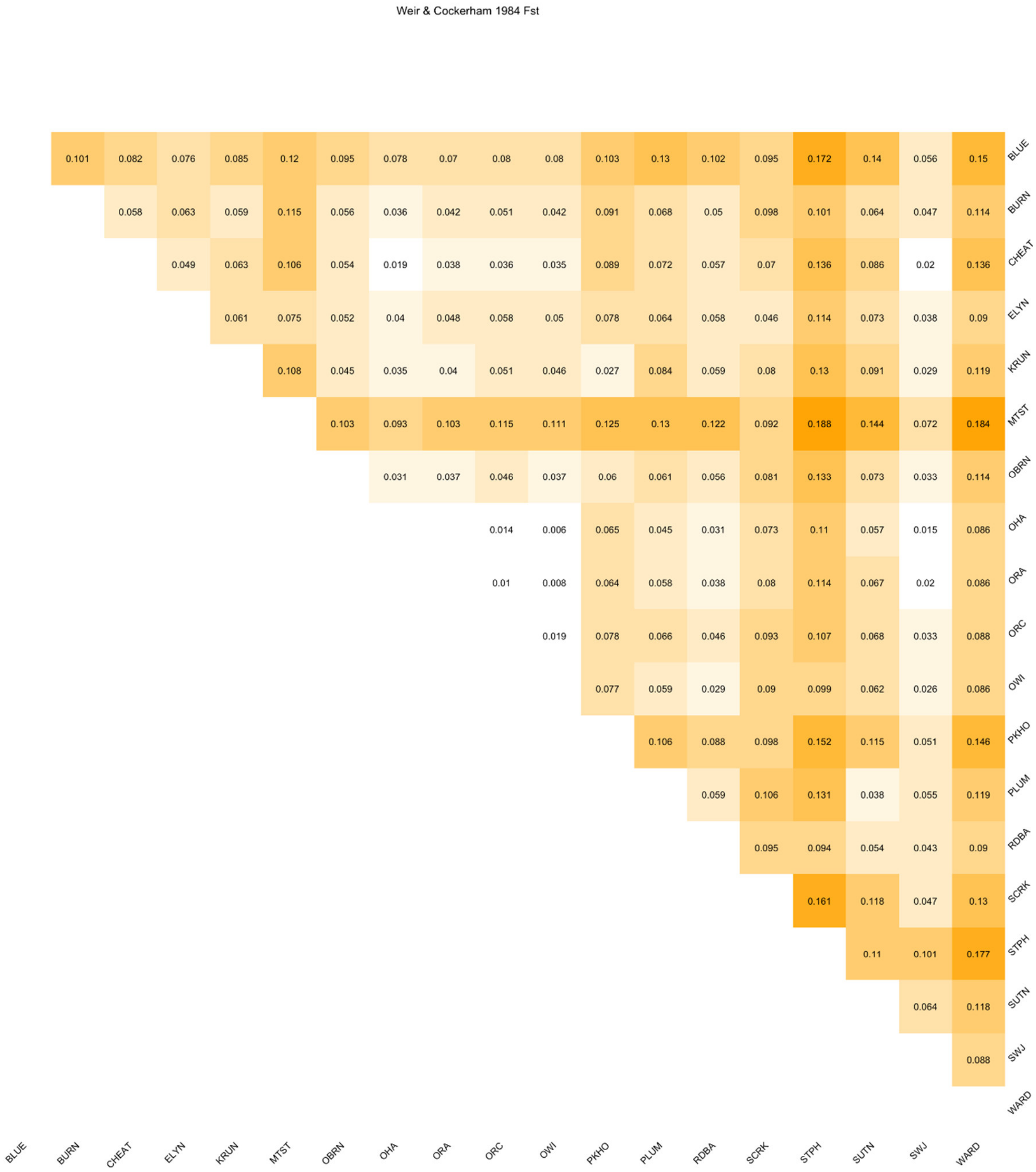


**Figure 6.** Pairwise $F_{ST}$ values between all sampled West Virginia largemouth bass populations using a total of 2,618 SNPs.

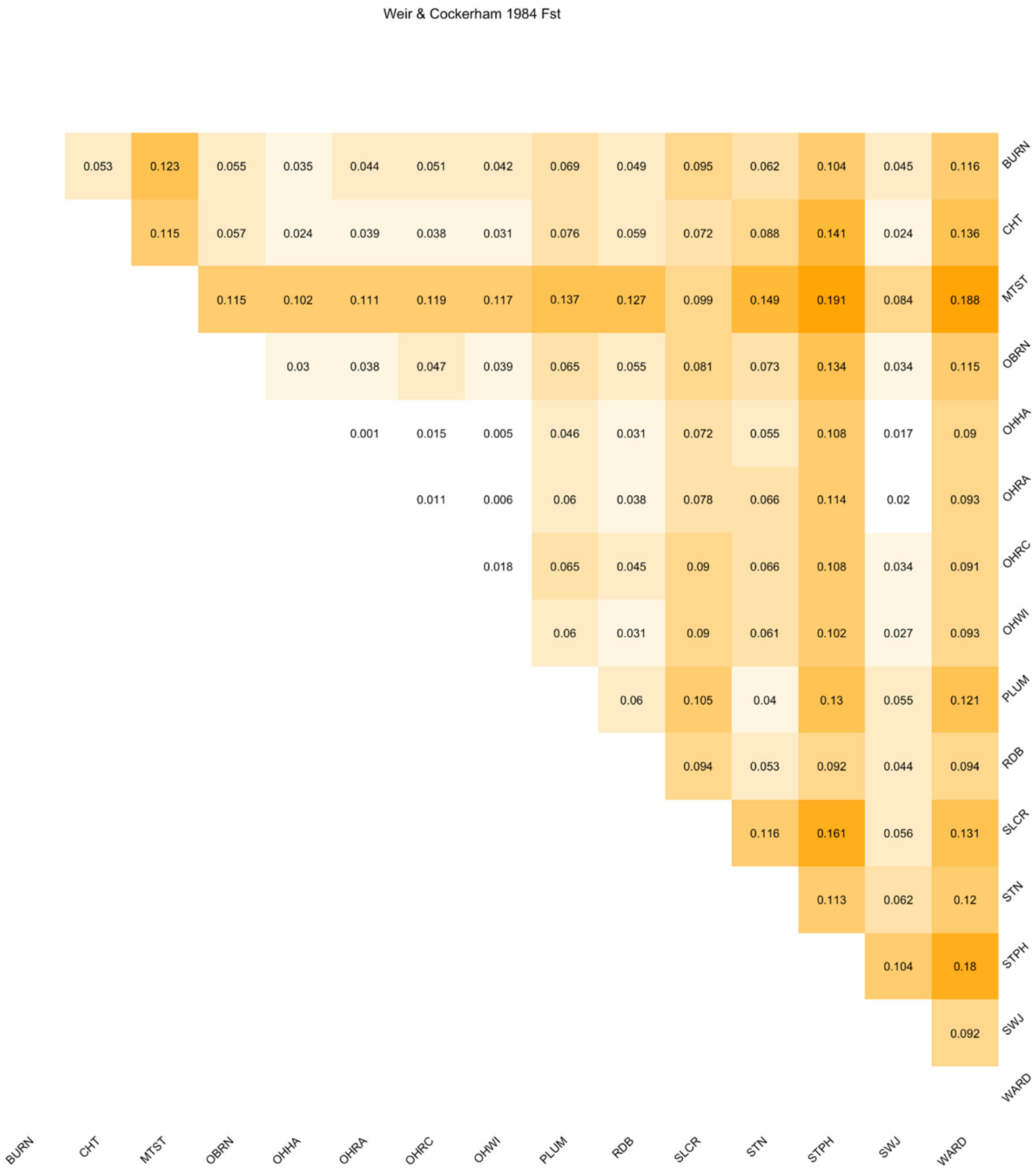


**Figure 7.** Pairwise $F_{ST}$ values between West Virginia largemouth bass populations dominated by the Ohio River strain ancestry using a total of 3,599 SNPs.
